# Estimating the Relevance of World Disturbances to Explain Savings, Interference and Long-Term Motor Adaptation Effects

**DOI:** 10.1371/journal.pcbi.1002210

**Published:** 2011-10-06

**Authors:** Max Berniker, Konrad P. Kording

**Affiliations:** Department of Physical Medicine and Rehabilitation, Northwestern University and Rehabilitation Institute of Chicago, Chicago, Illinois, United States of America; University College London, United Kingdom

## Abstract

Recent studies suggest that motor adaptation is the result of multiple, perhaps linear processes each with distinct time scales. While these models are consistent with some motor phenomena, they can neither explain the relatively fast re-adaptation after a long washout period, nor savings on a subsequent day. Here we examined if these effects can be explained if we assume that the CNS stores and retrieves movement parameters based on their possible relevance. We formalize this idea with a model that infers not only the sources of potential motor errors, but also their relevance to the current motor circumstances. In our model adaptation is the process of re-estimating parameters that represent the body and the world. The likelihood of a world parameter being relevant is then based on the mismatch between an observed movement and that predicted when not compensating for the estimated world disturbance. As such, adapting to large motor errors in a laboratory setting should alert subjects that disturbances are being imposed on them, even after motor performance has returned to baseline. Estimates of this external disturbance should be relevant both now and in future laboratory settings. Estimated properties of our bodies on the other hand should always be relevant. Our model demonstrates savings, interference, spontaneous rebound and differences between adaptation to sudden and gradual disturbances. We suggest that many issues concerning savings and interference can be understood when adaptation is conditioned on the relevance of parameters.

## Author Summary

Trying to explain how humans adapt to new motor behaviors and retain them over time is a central focus in motor control. Many aspects of adaptation, including savings and interference, have proven difficult to explain in a coherent manner. Linear dynamical models have been successful at describing the observed increase in performance while subjects familiarize themselves with an experimental perturbation. Many aspects of these experiments however, remain unexplained. In particular, while subjects display the ability to remember new motor behaviors for long periods of time, these linear models cannot. In this work we extend our previous body-world model of motor adaptation by estimating the relevance of inferred world disturbances. When these parameters are estimated to be relevant, they are used (and motor behaviors are adapted), and when they are estimated to not be relevant they are stored (and motor behaviors are remembered without being lost). Our model offers explanations for many observations on motor adaptation, savings and interference.

## Introduction

There is a large body of evidence to suggest that the nervous system maintains internal representations of variables that are relevant to the production of movement [1], [2], [3]. Internal models allow us to make repeatable and reliable movements despite a highly variable world and body, and our noisy perceptions of them. Ideally, these internal models ought to distinguish between the properties of the body and world, a crucial ability when generalizing movements [4]. Such a representation requires many parameters to represent how to control the body when interacting with external objects in the world. This in turn implies that many parameters of both the body and the world need to be estimated.

When estimating changes in the many parameters necessary to describe the interaction of the body and the world, it seems sensible that some of these parameters will change rapidly, while others change more slowly. Consequently a number of recent studies have constructed linear time invariant models that model adaptation unfolding over multiple time scales [e.g. 5,6,7]. These models have explained a wide range of temporal adaptation and savings phenomena.

While many linear models can explain motor phenomena associated with rapid re-adaptation, they are limited in their ability to explain phenomena of even short-term adaptation, as in savings after “washout” trials [e.g. 8], let alone the long-term effects of adaptation. For instance, linear models predict that aftereffects should decay with the same rate behaviors are adapted to, in contrast with experimental evidence [9], [10]. Linear models also predict that once a disturbance has been removed, its influence on movement is de-adapted and completely forgotten. This is clearly not the case, and subjects retain the ability to compensate for previously adapted behaviors over long periods of time [11], [12], [13]. In summary, while there are clearly multiple time scales at work, a linear time invariant process is not capable of explaining motor adaptation.

Since neither washout nor intervening days delete motor adaptations, there must be some mechanism that guards newly adapted parameter values against de-adaptation when they are no longer relevant. Motor architectures that can guard entire forward and inverse models of limb dynamics by switching them on and off have been proposed [14], [15], [16]. However, it is unclear how these models can account for the patterns of apparently incomplete generalization observed experimentally [4]. What's more, these models do not make a distinction between the parameters of the body and the world, but rather estimate when an appropriate model of the coupled body and world dynamics is applicable. In contrast, we propose the nervous system should separately estimate the properties of the body and the world and when those individual parameters are relevant for control. For example, our estimate of a coffee cup's weight is only relevant while we are holding it and not after we have set it down on the table. Estimates of our arm's weight, on the other hand, are always relevant for limb movements. Conditioning on such obvious relevance the nervous system can know not to adapt estimates of the cup's weight unless we are holding it. Parameter relevance, however, is not always this obvious. If this relevance could be estimated, then the nervous system could guard newly adapted behaviors and later retrieve them when they are relevant again.

To examine this idea, we designed an idealized model for computing the probability of relevance, and then using this estimated relevance to adapt. In a previous study we proposed a statistical inference model for motor adaptation that estimated a large number of parameters for the body and the world [4]. In a different study we proposed that the nervous system constantly estimates the relevance of errors for motor adaptation [17]. Here we combine these two approaches. We assume that parameters associated with the body are always relevant, whereas world parameters are only relevant under specific conditions. If the probability of a parameter's relevance is high, then it is subject to adaptation. If not, the motor errors may be due to sensorimotor noise or changes in body parameters.

**Figure 1:**
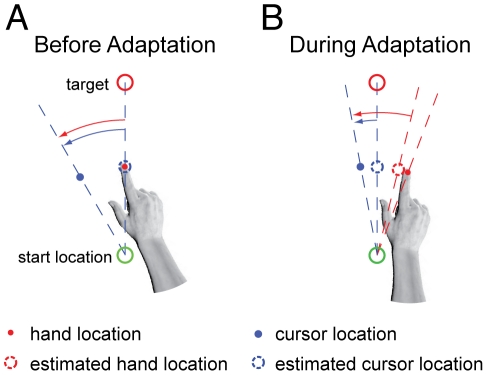
The likelihood of relevance. A) Before adapting, estimates for body and world disturbances are zero. The hand's path, along with the estimated hand and cursor location, fall along a straight path to the target. The large observed errors (red and blue arrows) of the perturbed visual display indicate the likelihood of a world disturbance being relevant is high. B) During adaptation, the hand's path is adjusted to compensate for the estimated body and world disturbances. Even though errors between the estimated cursor location and the observed cursor location have been reduced (blue arrow), the large error between the estimated hand location and the observed feedback (red arrow) continues to indicate the likelihood for the world parameter relevance is still high.

In contrast with the coffee cup example, the kind of experimental disturbances subjects are exposed to are not as evident. Therefore, we estimate relevance using a model that can predict the consequences of a class of world disturbances. As such, relevance defined here does not depend on a particular parameter value, but rather the particulars of that type of parameter's influence on motor behaviors. When movement patterns are consistent with a large world disturbance, regardless of the observed movement error (see Fig 1), then the likelihood of that parameter being relevant is high. For example, if the presence of a coffee cup in our hand, any coffee cup, can account for unexpected limb motions and forces on our hand, then parameters representing the cup's inertial properties should be subject to adaptation. If not, then those parameters should not be updated. In effect this allows for a rudimentary long-term memory, allowing for the retention and later retrieval of newly acquired world parameter values.

We simulated a series of experiments to investigate how our model behaves when adapting to multiple motor behaviors in succession. The model was restricted to four free parameters, which were held constant for all simulations. The models' predictions are consistent with the findings of savings, interference, spontaneous rebound and the differences between adaptation to gradual and abrupt disturbances. Our model offers a formalization of how the nervous system may estimate and store motor parameters when adapting to disturbances.

## Methods

### Generative model

The model used here is based on that used in a previous study [see 4 for details and code]. Briefly, the human upper limb is modeled as a nonlinear 2-link, 2 degree of freedom mechanism driven by feedforward torque components to compensate for estimated world and body dynamics, plus a feedback component to stabilize movements about a nominal, minimum jerk trajectory. For the results shown here only two parameters were inferred, a body-centric visuomotor rotation *θ_b_* (due to some possible combination of proprioceptive errors and relative head or torso rotations) and a world-imposed visuomotor rotation, *θ_w_*, the experimental disturbance of the cursor. The system observation, **y** *(t)*, is the visually observed (displayed cursor) position vector, **x** and velocity vector, *d* **x** */dt* of the limb's endpoint (or hand) in a Cartesian reference frame, **y** *=  [* **x** *(t), d* **x** *(t)/dt]^T^*. We assume this observation is corrupted by measurement noise, **n** *(t)*, with zero mean and covariance **R**.

We collate the parameters to be estimated in the vector, **p** *=  [θ_b_, θ_w_]^T^*. To infer these parameters, we assume that they vary according a random walk, with a small forgetting factor,



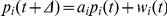



where *w_i_* is a zero mean random variable drawn from a normal distribution with variance, σ*_i_^2^*. These parameters influence the nonlinear dynamics of the limb, and the subsequent effects on movement are then observed in the output, **y**. However, we assume that influences of the world parameter, *θ_w_*, are only observed when the limb is perturbed. To denote this state of being perturbed by a visuomotor rotation, we define the relevance variable, *λ_rot_.* The variable can take on one of two values, one or zero. If a world rotation parameter is relevant then the relevance variable is one, if not, zero. The system's output then depends on the relevance in the following way,



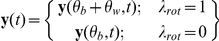



where **y** *(θ, t)* is shorthand for the observed output when visually rotated by *θ.* Though binary, we assume that the relevance parameter is also Markovian, and has a small non-zero probability of transitioning from one value to the other. We define a transition model (or mixing matrix), **M**  =  [0.999 0.001; 0.001 0.999], ensuring our prior probability of relevance never becomes fixed at 0 or 1. In total the model has 4 free parameters, *a_b_* and *a_w_,* σ*_b_* and σ*_w_*. However, to assure that estimates of the world would be retained over long periods of time, we held *a_w_* fixed at 0.99999.

### Estimating relevance

The model uses its observations of the limb's endpoint (**y**) to infer the probability that an external parameter is relevant and update its belief in the parameters in **p**. In this study we were focused on the interference and savings of visuomotor adaptations, therefore we limited our computations of relevance to the visuomotor variable, *λ_rot_*. Key in this computation is how the likelihood of relevance is computed. Before examining this, we first briefly describe how the posterior probability of relevance is computed using a Bayesian update. For ease of notation, we shall refer to the visuomotor relevance variable, *λ_rot_*, as *λ* for the remainder of this section. As defined above, *λ = 1* if it's variable is relevant, and *λ = 0* if not. The posterior probability that the world's rotation parameter is relevant, *P(λ(t) = 1|* **y** *(t))* is found through Bayes' rule,



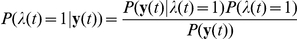



where *P(* **y** *(t)|λ(t) = 1)* is the likelihood (*P(* **y** *|λ = 1)* for brevity), and *P(λ(t) = 1)* is the prior (*P(λ = 1)* for brevity). Note that the prior is the posterior found in the previous time step, modulo the transition model, *m_11_ P(λ = 1)* + *m_12_ P(λ = 0)*, since it summarizes the probability of being relevant based on all the observations made up to that time (we assume *λ* is Markovian).

Our definition of relevance is based on a type of parameter's ability to explain disturbances. To illustrate, consider reaches early during adaptation. The body and world parameter estimates of a visuomotor rotation are zero and there are large movement errors. These errors are consistent with a large world disturbance and the probability of the visuomotor parameter being relevant is computed as high (Fig 1A). After adapting for some time, an updated estimate of the body parameter partially compensates for the disturbance. The newly estimated world disturbance further compensates for the disturbance. Any remaining errors are used to update these parameter values with a Bayesian update (see below). However, even if the errors are driven to zero, there remains a large apparent error between the observed movement, and how much the body parameter can account for (Fig 1B). If this mismatch can be explained as the result of a relatively large world rotation, then the likelihood of a world disturbance is high. Therefore the corresponding likelihood of relevance is based on the probability of observing the cursor (**y)**, given our current estimate of the body parameter, and a world rotation of any value perturbing our observations. To compute this we must integrate the probability of a perturbed observation over all possible world rotations,



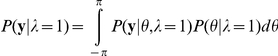



*P(* **y** *|θ,λ = 1)* is the likelihood of observing the limb's endpoint with a given rotation, *θ*. Since body parameters are always relevant, this likelihood is a normal distribution centered on the internal model's prediction, N(**y** *(θ+θ_b_)*, **R**). Rather than integrate this distribution over the forward model's prediction over each movement and all possible world rotations we made the following simplifying assumption. Since the visuomotor disturbance influences movement observations in a relatively simple and unique manner (a constant rotation), we redefined this likelihood using only visuomotor angles. We used the hand trajectory, to identify the unique rotation, *θ_y_*, that minimized the root mean squared error between the observed limb path, **y** and the estimated path when only compensating with the, always relevant, body estimate. We then use a Normal distribution over *θ* centered on *θ_y_*, with the variance associated with an observation of the rotated limb, σ*_θ_^2^* (see below). Although the normal distribution is defined over all real numbers, the variance of this distribution is much smaller than our limits of integration, and can very accurately be described as restricted between − *π* to *π*.

To define the prior over visuomotor angles, *P(θ/λ = 1)*, we note the following considerations: relevance is based on the ability of any visuomotor disturbance to explain the data, and we want to avoid biasing the inference. The prior should be flat over all non-zero rotations, but avoid assigning high probabilities to the degenerate case of small (relative to our observation noise) or zero rotations. Based on these considerations we defined the prior as *(1-exp(−θ^2^/2σ_θ_^2^))/Z* where *Z* is an appropriate normalizing constant. Just as above, given that the variance for the Gaussian term is much smaller than the domain, 2*π*, *Z* is very accurately approximated as 2*π* 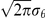. This form of a prior assigns high probability to all large valued rotations, and low probability to rotations that are near zero, or small relative to the size of the observation noise, σ*_θ_^2^*.

After integrating the above equations we find an expression solely in terms of the rotation that corresponds to our observation, *θ_y_*,



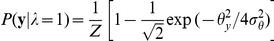



To summarize, this likelihood assigns high probability when the observed rotation, *θ_y_* is large relative to the observation noise. We also note that in this idealized model, *θ_y_* is the angular displacement relative to a movement predicted using the current estimate of a body disturbance, *θ_b_*. Thus estimated body disturbances influence the forward model's belief of where in space the limb is. Finally, we also need to compute the likelihood of the unperturbed condition, *λ = 0,*



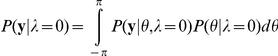



We can define *P(* **y** *|θ*,*λ = 0)* with a Normal distribution just as before. However, since this is for the case when the rotation is not relevant, this distribution should be centered on *θ = 0.* The prior, however, will be different. The prior should only assign large probability to rotations that are small, or small relative to the size of the observation noise. Therefore we define the prior as a Normal distribution with zero mean and variance, σ*_θ_^2^*. Again, since the variance for these distributions is very small relative to the limits of integration, both the likelihood and prior can be accurately approximated as restricted between −*π* to *π.* After integrating, we arrive at



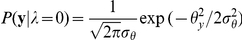



With this final term found, we can express, *P(* **y** *) =  P(* **y** *|λ = 1) P(λ = 1) + P(* **y** *|λ = 0)P(λ = 0)*, and compute both the posteriors, *P(λ = 1|* **y** *)*, and *P(λ = 0|* **y** *).*

The variance, σ*_θ_^2^*, was found by noting that the angle subtended by the arm's length, L, and one standard deviation of the observation noise in either direction, is approximately 2σ/L, where σ is 0.01 meters. Using either the upper or lower arm length for L, the angle is approximately 1.7°. Using the whole arm length for L, the angle is 3.4°. Therefore, we defined σ*_θ_^2^* =  (2.5 *degrees*)^2^. During the error clamp simulations the model's observation was artificially constrained to have zero error, regardless of the parameter estimates used to generate motor commands, or their relevance. The model's observations of movements that attempted to compensate for disturbances were no different from estimated movements without disturbances. To model this uncertainty, we held the likelihood fixed at 0.5 during these circumstances. We note that denying the model the evidence necessary to compute a likelihood (as may occur in error clamps) also has the same effect, as the transition matrix relaxes the probability of relevance to 0.5 as time passes.

### Optimal inferences

With the relevance probabilities in hand, we can then infer estimates of the parameters. The estimate of the world's rotation used by the model to make predictions and compute commands is conditioned on the prior probability of being relevant,



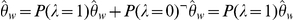



since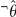 (the rotation when not operating in a visuomotor rotation) is assumed to be zero. This expected world estimate along with the body estimate is collated in the vector 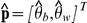.

If the probability of relevance is one, then the update for the rotations is the extended Kalman filter update,



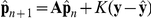



where **A** is a matrix with *a_b_* and *a_w_* on the diagonal. However, if the probability of relevance is zero, then the world rotation is guarded against adaptation, and the update is



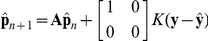



Therefore, we approximate the update with the maximum likelihood update,



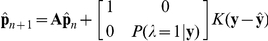



The parameters' covariance, **P**, was updated in a similar fashion. Defining **P** *_n+1_ =* **AP** *_n_* **A** *^T^ +* **Q**, and the updated covariance 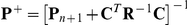, then the posterior covariance was approximated as



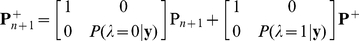



We note that multiple approximations to the updates for the parameters and their covariance were attempted and the qualitative results did not change. Furthermore, the transitions from low to high relevance are relatively quick (2–3 trials). As such, the approximations for inference during the intermediate state of relevance/non-relevance (*0<P(λ = 1)<1*) have only a limited influence on the estimated parameter values.

### Simulations

The limb parameter values were based on [18]. For all simulated experiments, the targets and reaching distances were equivalent to that used in the studies. For all simulated movements we assumed the nominal limb trajectory was that of a minimum jerk profile specified by the target locations, via points (8 equally spaced locations) and movement times reported. Parameter estimates were updated 6 times per movement, and movement targets were randomly selected. The probability of relevance was computed once per movement. The three free parameters, *a_b_*, σ*_b_* and σ*_w_*, were tuned by hand to create qualitative fits to the data from [19]. These values were then used for the remaining simulations.

Our simulated visuomotor experiments display trial-by-trial adaptations, whereas experimental plots of the same data are of cycles (data averaged over 8 consecutive trials). We have not made a distinction between trials and cycles because of the rescaling properties of the inference process. A single trial in our formulation need not represent a single trial or a cycle. The model is time invariant in this regard and we can scale all the parameters (jointly) to scale time by any specific value.

## Results

### The sources of motor errors and their relevance

In our previous model [4] parameters were always relevant and subject to adaptation. For variables that describe the body this makes intuitive sense. Variables that describe the environment, however, may only be relevant in a particular circumstance [17]. We thus amended the source estimation model, partnering world parameters with relevance variables. The probability of being relevant is found by comparing the observed movement with the movement predicted if the estimated world disturbance were neglected. The estimate of a world parameter is then adapted using a Kalman update weighted by the probability of being relevant (see Methods). This contextualization allows for the storage and later retrieval of newly acquired parameter values.

In this study we focus on the paradigm of visuomotor adaptation, restricting the model to estimate two variables, a body-centric visual rotation (e.g. a rotation of the head relative to the torso and/or arm) and world-imposed rotation (the experimental manipulation). As a result, the model can only entertain one visual disturbance due to the body and one due to the world. We restrict the model to four free parameters: two parameters to describe the magnitude of noise associated with them, and two decay rates or time scales. However, we further assume the decay rate for world parameters is essentially zero, allowing for the long-term retention of that estimate. The existence of a fast and slow time scale are consistent with previous findings [5], and our previous work [4] which suggests the uncertainty associated with body parameters is large, and estimates should vary quickly. The resulting model offers predictions for how adaptation should proceed when it is statistically optimal.

Though the relevance model we present here is nonlinear in both the limb dynamics and the adaptation scheme, the results we present share many similarities with those of previously published linear models of adaptation. Specifically, when adapting to a visuomotor rotation of the model's hand location the motor errors appear linear in the estimated disturbances. Furthermore, although these disturbances are not adapted with a fixed rate (but instead estimated with an extended Kalman filter), trial-by-trial changes in the estimates are small and the resulting motor errors follow typical exponential trajectories. Due to these similarities the relevance model has the appearance of a linear estimation process with a nonlinearity that switches the estimated world disturbance in and out of the adaptation process.

### Short-term adaptation and savings

To examine short-term motor adaptation, many experiments expose subjects to a disturbance twice in quick succession, with either a counter disturbance or a washout period in between. Savings are observed on the second presentation of the disturbance in both cases. Linear models can explain savings after adaptation in the form of an increased learning rate when adapting to the counter disturbance paradigm [5], [6]. However, linear (time invariant) models are not capable of explaining this same type of savings after a sustained washout period [8]. Once the perturbation has been removed, the model necessarily de-adapts its parameters. Therefore, a washout period lasting as long as the adaptation period would reverse any savings; a second exposure to the disturbance would proceed just as the initial one. Without a mechanism for guarding parameters against de-adaptation, linear models are incapable of displaying even this form of short-term motor adaptation.

**Figure 2:**
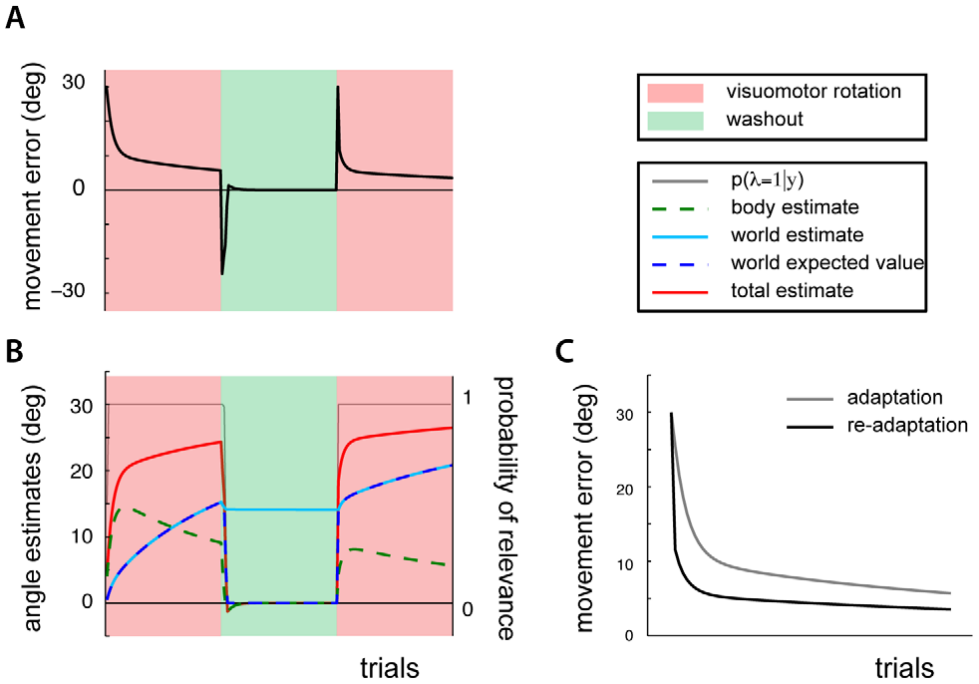
Short-term savings after washout. A) Angular reach errors during the first presentation of a visuomotor disturbance, washout (while grasping robot) and subsequent presentation of the same disturbance B) Inferred body and world rotation parameters during adaptation and the corresponding probability of relevance. C) Angular reach errors from first and second presentation of visuomotor disturbance overlaid.

Consider how the model presented here adapts while making reaches with a visuomotor perturbation. Initially the model cannot predict the consequences of, nor compensate for, a visual disturbance, and there are large motor errors (see Fig 1A, 2A). These errors drive adaptation of the estimated body rotation. At the same time, the model estimates that a large angular rotation of the hand's path is consistent with the observed reach (Fig 1A). This large potential angular perturbation indicates that the probability of the world's visuomotor rotation relevance is high (approximately 1, Fig 2B). As a result the world's rotation estimate is adapted and rises to help compensate for the experimental perturbation (Fig 2B). Although the motor errors progressively decrease, the model is still aware that a large visuomotor rotation is consistent with the ongoing observations; there remains a large discrepancy between the observed reaches and the model's estimate of an uncompensated reach. An estimate of the uncompensated reach is found by predicting a reach made without compensating for the estimated world rotation. The estimated body rotation however, is still used, and biases this estimate (see Fig 1). A large angular perturbation continues to be estimated and the probability of relevance remains high throughout the adaptation process. After an adequate number of trials, the contribution from the body and world rotations largely cancels the visual disturbance and the errors are small (Fig 2A). The overall motor behavior is qualitatively consistent with adaptation to a novel visuomotor disturbance. Both linear models and our nonlinear model can correctly describe the resulting patterns of adaptation.

Continuing with the short-term adaptation paradigm, when washout trials are subsequently presented, consistent with experimental findings, the relevance model produces large motor errors in the opposite direction (Fig 2A). The model, now biased by its previously adapted body rotation, mistakenly estimates an angular perturbation now in the opposite direction. The probability that the world's visuomotor rotation estimate is relevant remains high and both the body and world estimates de-adapt (this produces a short lasting overshoot in the error, Fig 2 green panel). As the body estimate quickly de-adapts the probability of relevance decreases back to zero. This change in the world's estimated relevance halts adaptation of the world rotation parameter. In contrast with similar linear multi-rate models, the motor errors are now only used to estimate the body's rotation parameter (which is always relevant). The body's estimate continues to de-adapt and the motor errors vanish. This combination of the fast change in the world parameter's relevance, along with the fast adaptation rate for the body parameter, results in the relatively quick de-adaptation back to nominal reaches. When the disturbance is turned on again, large errors result. Just as before, the probability that the world's visuomotor rotation parameter is relevant increases. This quick change in the estimated relevance results in a relatively fast decrease in errors, as the world's rotation estimate begins to compensate for the rotated reaches (Fig 2C). Thus the estimation of relevance allows the model to explain fast re-adaptation.

### Long-term savings and interference

The adaptation and short-term savings we have reviewed above have similar analogues over longer time frames. To examine savings over multiple days, subjects adapt to a disturbance, and then are presented with the same motor disturbance on a subsequent day. In another paradigm, subjects adapt to two disturbances in quick succession, the later often a counter disturbance, and then evidence for savings or interference is examined on a subsequent day [e.g. 13,19]. Both experimental paradigms demonstrate that many of the features of short-term motor adaptation also exist over longer time frames. Unfortunately linear models are not capable of describing some of these phenomena over these longer time frames.

**Figure 3:**
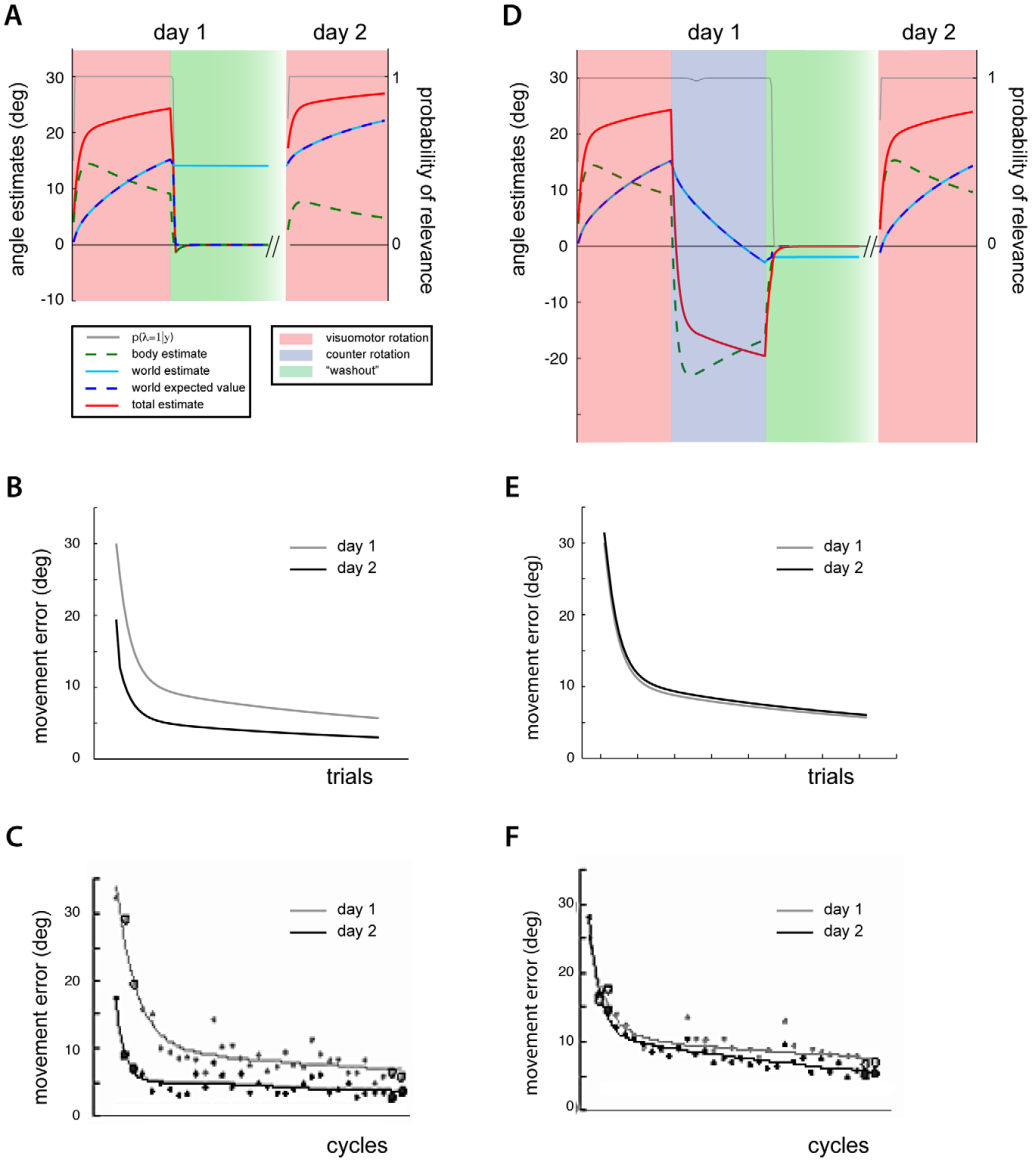
Long-term savings and interference. A) Inferred body and world rotations and the corresponding probability of relevance during the first presentation of a visuomotor disturbance, washout (after experiment has ended) and subsequent presentation of the same disturbance on a second day. B) Angular reach errors from the first and second presentation of the visuomotor disturbance overlaid. C) Experimental findings after the same adaptation (reproduced from [19]). D) Inferred body and world rotations and probability of relevance during a visuomotor disturbance, an oppositely oriented disturbance, washout (after experiment has ended) and subsequent presentation of the original disturbance on a second day. E) Angular reach errors from the first and second presentation of disturbance overlaid. F) Experimental findings after same adaptation (reproduced from [19]).

Using the relevance model to examine its predictions for saving over days, we simulated these experimental paradigms [e.g. 19]. No changes were made to the model for these long-term adaptation results. The first day's adaptation to a visuomotor disturbance proceeds just as described above (Fig 3A). There is a subsequent washout period just as before, where the probability that a world rotation estimate is relevant quickly decreases towards zero. The world's parameter estimate, no longer relevant, is left for later use should it become relevant again. The body estimate then rapidly de-adapts (Fig 3A).

In this particular experiment, subjects do not undergo a period of washout with the robot, but instead leave the experimental setting. Therefore this washout is different from those of the previously described short-term experiments on two counts. First, the washout trials predicted by the model correspond to natural movements made by the subjects after the experiment has ended. Our model thus predicts that there should be aftereffects that persist after the subject has let go of the robot handle. This is consistent with recent evidence [e.g. 20]. Second, since this washout period does not take place while grasping the robot handle, the last interactions subjects have with the robot are associated with a disturbance; the robot is an unambiguous proxy for the relevance of a visuomotor rotation. Therefore we assume that the model's initial probability of the visuomotor parameter's relevance should be similar when the model next returns to the experiment.

When adaptation on a subsequent day is simulated, the probability of the visuomotor parameter being relevant is initialized to a high value (0.75), as discussed above, and the world's rotation estimate is believed to be relevant. The model is again presented with the same visuomotor rotation and the initial motor errors are lower than those of the previous day (Fig 3B). At the end of this second day of adapting to the visuomotor disturbance the movement errors are lower than on the previous day. This is due to the relatively large contribution from the world estimate. The model's predictions for long-term savings are consistent with the observed experimental findings (Fig 3C).

We simulated the second experimental paradigm, now presenting two visuomotor rotations of opposite orientations in succession [19]. Adaptation to the first motor behavior proceeds just as above. When the model is presented with a second, oppositely directed rotation, the model again estimates a large angular discrepancy between the observed hand path and an estimated path that neglects the current world rotation estimate. However, this estimated rotation of the hand's path is now in the opposite direction. Regardless, the probability that the visuomotor rotation parameter is relevant remains high. Both the world and body parameters begin adapting to a rotation with the opposite sign (Fig 3D). When the washout trials begin, the probability of relevance quickly decreases, the world's rotation estimate is no longer used, and adaptation is halted. However, by this point all adaptation to the first visuomotor rotation has largely been lost. Just as above, on a subsequent day the model begins with a belief in a visuomotor rotation's relevance, and uses its estimated world rotation. Yet, the small estimate has little influence on the movements. Consistent with experimental findings of interference, the model performs as if naïve on the second day's presentation of the disturbance (Fig 3E, F).

Our new model explains long-term savings in the form of retention of a previously adapted motor behavior and decreased initial errors. Further, the model demonstrates how adaptation to two similar disturbances can cancel each other's influences and result in interference. Both findings are widely observed in motor adaptation studies.

### Adaptation to sudden and gradual perturbations

Most studies examine adaptation after the sudden introduction of a perturbation. However, recent evidence has found marked differences when subjects adapt to a perturbation that is gradually introduced. These gradually introduced perturbations have been used to examine both interlimb generalization, and savings of motor behaviors across multiple days. In one study, subjects adapted to a force field that was either suddenly or gradually introduced [21]. After adapting, savings were examined when making test reaches with the non-dominant limb in the same force field (at full strength). The test reaches made after adaptation to the gradually introduced perturbation exhibited relatively larger deviations from a straight path. The initial errors were roughly twice as large as those found after adapting to the suddenly introduced perturbation, suggesting generalization of the adapted force field to the other limb was relatively poor when the perturbation is gradually introduced. In another study examining the differences between gradually and abruptly introduced force fields, post-adaptation reaches made without grasping the robot handle were examined [22]. The aftereffects on these free reaches were larger when subjects adapted to a gradually introduced perturbation. This suggested adaptation to a gradually introduced force field, may have altered the way subjects controlled their limb. Another study examined savings across days with a visuomotor rotation that was either gradually or suddenly introduced [23]. After adapting on one day, subjects made reaches in the same visuomotor perturbation (full strength) on a subsequent day. Subjects that had adapted to the gradually introduced perturbation made slightly larger errors initially, even though they adapted over more trials than the other group. These three results, and other studies like them, with their distinctions in savings, may offer testable predictions for how the nervous system adapts.

**Figure 4:**
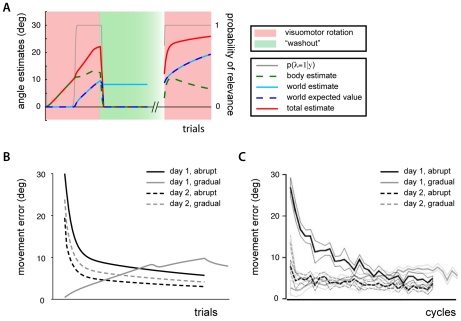
Savings after a gradually introduced disturbance. A) Inferred body and world rotations and probability of relevance while a disturbance is gradually introduced, washout (after experiment has ended) and presentation of full disturbance on a second day. B) Angular reach errors on first and second day after adapting to a gradually (grey lines), or suddenly (black lines) introduced disturbance. C) Experimental findings of same adaptations (reproduced from [23]).

To examine our model's predictions we simulated the same gradual perturbation as the one used in [23]. During the early trials the motor errors are small and the body estimate quickly adapts to them. Because these errors are small the body estimate does an adequate job of compensating for the perturbation. The model does not detect a large angular perturbation and does therefore not believe the world's rotation estimate (initially zero) to be relevant. Only during later trials as the perturbation strength increases does the model believe the world's parameter is relevant. Thus, much of the adaptation is accounted for by the body estimate (Fig 4A). After the simulated experiment has ended, the model has a world estimate that is little more than half as strong as would be otherwise (compare with Fig 3). Our model predicts three findings of interest. First, we can conclude that during a generalization trial with the other limb, the model's errors would be approximately twice as large as if the perturbation was suddenly introduced, consistent with experimental evidence [21]. Second, because the perturbation is largely attributed to the body, the model predicts relatively large aftereffects during reaches made without the force field, when the robot handle is not grasped and the probability of a disturbance parameter's relevance is zero [22]. Third, since the world estimate of a rotation is smaller than would be otherwise, movement errors on a subsequent day are larger initially, just as was found experimentally (compare Fig 4A, B). Our model thus provides an interpretation of the effects that are associated with fast versus slow introductions of perturbations.

### Error clamp adaptations

One additional set of phenomena may be important to characterize the properties of motor adaptation. In several recent studies subject's motor behaviors are examined when they make reaches in an “error clamp”, or “force channel”, wherein force disturbances are removed and movements are constrained to be straight. This is done to examine how and if subjects alter their motor strategies in the absence of kinematic errors. In an early study, after subjects adapted to a velocity-dependent force field, an error clamp was unexpectedly turned on [24]. Even though there was no longer any need to compensate for the force field, subjects continued to produce considerable forces as if it were still present. These forces slowly decayed, over a longer period of time than the subjects required to adapt or de-adapt in the absence of an error clamp. This suggested that these erroneous forces and their slow decay were the result of some altogether different process.

**Figure 5:**
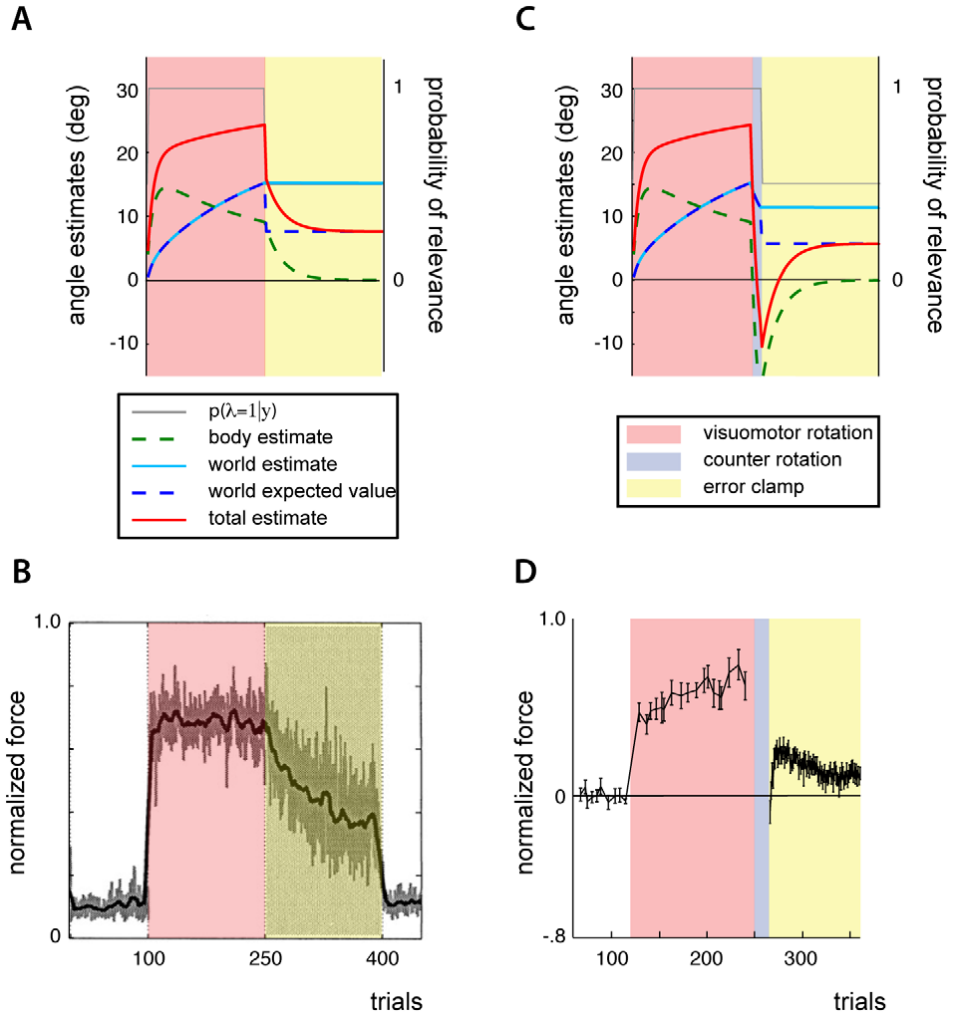
Error clamps and spontaneous rebound. A) Inferred body and world rotation parameters and probability of relevance during adaptation to a visuomotor disturbance and subsequent error clamp. In the error clamp, feedback indicates a lack of errors regardless of movements. B) Experimental data of normalized reaching forces during adaptation to a force disturbance and subsequent error clamp (reproduced from [24]). C) Inferred body and world rotations and the probability of relevance during presentation of a visuomotor disturbance, visuomotor disturbance of opposite orientation and subsequent error clamp. D) Experimental data of normalized reaching forces during a force disturbance, opposite disturbance and subsequent error clamp (reproduced from [5]).

We can examine what the model would predict by simulating similar circumstances. The model is first presented with a visuomotor rotation, and then the reaches are “clamped” to constrain movement errors to be zero. Adaptation proceeds just as we have seen before (Fig 5A). Under the simulated error clamp condition, regardless of what the model (or subjects) does to compensate for a perceived disturbance, they observe the same error-less outcome. The model cannot observe the consequences of using its estimated perturbations; this results in uncertainty in the relevance of the visuomotor parameter (see Methods). As a result the model partially uses the world's estimate to compensate and both the body and world rotation estimates slowly decay towards zero. The results are qualitatively similar to experimental findings (Fig 5B).

In a somewhat different paradigm, after subjects adapt to one disturbance they are briefly presented with a counter disturbance and subsequently make reaches while errors were clamped [5]. Under these circumstances subjects temporarily make reaches as if they are compensating for the counter disturbance, even though it is not present. This phenomenon, termed spontaneous rebound, has been observed under a variety of conditions [25], [26], [27]. Ideally models of motor adaptation should be able to describe such a behavior.

How would the source relevance model explain such findings of spontaneous rebound? We can simulate the model's predictions to the same paradigm with a visuomotor rotation first, then a counter rotation, and then a “clamp” where we artificially constrain the movement errors to be zero. The model can predict spontaneous rebound through the interaction of two mechanisms. As with other linear multi-rate models there is the interaction of two or more processes with different adaptation rates [e.g. 5]. But more importantly for our model, under the simulated error clamp condition, the model (and subjects) observes an error-less outcome, regardless. This results in uncertainty in the relevance of the visuomotor parameter and the model partially uses the world estimate. The model appears to overcompensate for a nonexistent rotation and the results are similar to experimental observations (Fig 5C, D). Though other multi-rate models can explain spontaneous rebound, our model offers a different explanation in terms subject's difficulty in gauging the circumstances under which they are adapting.

## Discussion

Here we have extended a body-world, multi-rate model to infer not only the parameter values but also their relevance to the current motor conditions. The discrepancy between observed movements and those predicted when neglecting world estimates is used for the computation of relevance. World parameters that are estimated as having little relevance are not used to generate motor commands and are not adapted. Body parameters, however, are assumed to always be relevant and subject to adaptation. In effect, this allows for a rudimentary long-term memory of world parameters, allowing for the retention and later retrieval of newly acquired parameter values. The entire process is dynamic and requires no intervention for describing behavior across short or long time frames. We have demonstrated that such a model can explain a wide range of findings on human motor control. Our results are consistent with the basic findings of savings and interference, error clamp results, and the differences between adapting to gradual and abruptly introduced disturbances.

Though there are some clear similarities between the model we present here and other computational descriptions of motor control and adaptation, there are important distinctions. Our model makes a categorical distinction between parameters that represent the body and those that represent the world; thus it shares similarities with two-rate models [5], [6]. Indeed, our model makes nearly identical predictions for short-term savings, interference and reduced learning rates with increased adaptation duration [28]. Since these models are linear, however, they cannot explain adaptation on longer time scales, as all their adapted parameters relax back to zero. Perhaps a more fundamental distinction, it is not clear what the “fast” and “slow” variables in multi-rate models represent computationally, although they may be related to distinct neural structures at the implementation level. The model we present offers explanations for a range of findings on both short and long-term motor adaptations as well as generalization [4]. Further, we model the estimation of body and world variables that can be tested through future experimentation.

Since our model switches the world parameter values in and out based on their probability of relevance, it bears some resemblance to the other models that switch modules on and off, such as the mixture of experts and MOSAIC [14], [15], [16]. However, our representation of world and body parameters within a dynamical model is distinct from the MOSAIC controller's modules of paired forward and inverse models of whole body-world dynamics. The MOSAIC controller does not independently represent the body and the world (which is a cornerstone of our model). In fact, even if the MOSAIC were altered to represent the body and the world in two different modules, they could not be “summed” to represent whole body-world dynamics, as these descriptions are coupled and highly nonlinear. Our proposed model represents distinct parameters within a model of the limb and body dynamics. Therefore it can uniquely adapt these parameters, and use them for generalization in a manner MOSAIC cannot.

Furthermore, our use of a relevance parameter is distinct from the notion of context used in these switching controllers. In the MOSAIC model, modules are switched on and off based on the similarity between their predictions and the observed motor behavior. Each module's predictions are uniquely described by the current parameter values that make up that module (e.g. its current estimate for a visuomotor rotation or force field). As a result, a module for a particular force field will not be switched on unless the limb makes reaches in a very similar force field. Our computation of relevance is based not on a parameter's value, but on the manner it influences motor behaviors. For example, the parameter for an inertial perturbation is likely whenever limb movements are consistent with an inertial perturbation of any sufficiently large value.

In large part due to these differences in relevance and context, it is not clear if the MOSAIC model could also explain some of the findings we have presented here. For instance, consider adapting to a 30° visuomotor rotation. A module representing the perturbed limb dynamics would modify its parameters to compensate for the disturbance. When a −30° rotation is then presented, this module's prediction errors (now ∼60°) would in fact be larger than a baseline, null condition module (only ∼30°). As a result the context variable for the module associated with the visuomotor rotation would be switched off, and this module would not continue adapting to the counter rotation; the model would not predict interference. Through a similar line of reasoning it is not clear how the MOSAIC model could explain the phenomena of spontaneous rebound.

Other studies have used the idea of context in different ways. In one study context was defined as the implicit memory of the limb segments used during a motor behavior [29]. In a sense, this assigned relevance to different body effectors. In a more recent study context indicated visuomotor rotations of different magnitudes [6]. In those studies context was known unambiguously, not estimated based on errors or changes in the environment, as we have done here. Further, here we define relevance (similar to context) in terms of the existence of external disturbances, regardless of what limb segment is used or the strength of the particular magnitude of the disturbance. Our study can thus be seen as a generalization of these studies to unobserved contexts and changes in the environment, which makes new experimentally testable predictions about the role of relevance.

In this work we have examined the effects of adapting to a visuomotor rotation, however, this model could be extended to adapt to other types of disturbances as well. In particular, several experimental studies have investigated how adapting to visuomotor rotations and altogether different motor disturbances in quick succession, effect interference and savings [19], [30], [31]. Interestingly, the results of these studies, having contrasting findings on savings, have motivated distinct interpretations concerning the nervous system's ability to represent kinematics and dynamics uniquely. Within that context, these results were argued to be incompatible. Our model makes no distinction between kinematics and dynamics but instead a distinction between parameters that represent how to control the body and how to interact with the world. Furthermore, our model predicts that if the effects of two different perturbations were similar (in terms of their resulting motor errors and sensory consequences) then their accompanying world estimates (e.g. estimated world rotation, or estimate world force field) would both be assumed relevant for adaptation. Therefore, adapting to a visuomotor rotation, and then a force field that perturbed the limb in a similar manner, might produce interference [19], whereas the subsequent adaptation to a force field dissimilar to a visuomotor rotation wouldn't [30]. As such future work using this model may offer a unique perspective to examine the findings of these and similar experimental studies.

For the sake of focus and instruction, we have modeled one estimate per disturbance, i.e. one estimated world rotation. This assumption played a crucial role in some of our findings on interference. If instead we had allowed for multiple estimates of a world rotation, it is not clear how the model would predict interference when modeling adaptation to counter disturbances across multiple days. Indeed, other studies have found that under appropriate conditions, a newly acquired motor behavior can be consolidated and resist retrograde interference [32], [33]. Our model does not predict these findings but extensions that could also explain these effects would be interesting. Such extensions might be possible by introducing parameters to describe multiple visuomotor disturbances, each with their own uncertainty. Such a model could implement a form of supervised adaptation; after adapting to, or operating within, a specific visuomotor disturbance for a long time the model could grow certain of this parameter value. Then, adapting to a similar but oppositely directed disturbance would require adapting another, less certain, visuomotor parameter. Such a scheme might implement adaptation to multiple disturbances, consistent with the idea of consolidating a motor behavior and learning a second, distinct behavior without interference.

We feel that much if not all of the model's value lays in the intuition it yields in trying to explain motor behavior phenomena. The studies and accompanying simulated results we present are those that we feel the model may help to explain. However, as with all models, this model is necessarily false [34], and there are experimental findings the model either cannot explain or that are flatly at odds with its predictions. For example, though our model is consistent with the findings on adapting to gradually versus abruptly presented perturbations in the Klassen et al. study, a more recent examination found distinct results. In this new study rates of motor decay were probed during short-term adaptation to a force field, either abruptly or gradually introduced [35]. Though the aim and experimental protocol of this study was very different from the Klassen study, some apparent contradictions were found in that there were no effects on the re-adaptation to the force field between the abrupt and gradual groups. To be clear, this finding was made under conditions of short-term savings of a force field (not long-term retention of a viruomotor disturbance), and obtained with the use of error clamps. However, despite their differences, it is not obvious to us how our model could account for these two distinct findings.

In contrast with the gradual vs. abrupt findings presented above however, our model makes an interesting prediction that could readily be tested. According to our model the amount of adaptation for world parameters is due to both the size of disturbance and the amount of training; the larger the disturbance and the more training time, the more a world parameter is adapted. Similarly, the more world parameters are adapted, the more savings should be observed on a second day's presentation of the disturbance. Surprisingly though, our model predicts that even with a gradually introduced perturbation, and one that never reaches the strength of the abruptly presented one, more savings can be observed on a second day. If the model is presented with a visuomotor rotation that is ramped up slowly over many trials, the world estimate will have relatively more time to adapt, and the body estimate more time to de-adapt. As a result, even if adaptation ends before the visuomotor disturbance has reached, say 30°, the world estimate will surpass that seen in the abruptly presented paradigm. Thus more savings, not less, will be observed on the second day. The results of such an investigation would be very informative for the study of adaptation.

Another study of force field adaptation offers both supporting and contradicting evidence for our model. In this study the rates at which subjects adapted (as quantified through movement errors) were compared when adapting either to the null field or a scaled down version of the force field [9]. Consistent with our model, de-adapting to the null field is much faster than adapting to the force field. It was also found that subjects adapted to the scaled down force field even faster than they did the null field. In contrast with this finding, our model would predict that both the body and the world would adapt to the scaled down force field, resulting in a relatively slow process. This is in sharp contrast with their findings and will provide an interesting target for future modeling efforts.

In our model we have assumed that movement predictions always utilize body estimates. Since the body is always relevant, this seems sensible. One consequence of this is that the model is in effect “blind” to changes it has inferred are due to the body; the model cannot make predictions for movements that do not compensate for these adapted body estimates. Even if the inferred body estimates are due to an experimental perturbation, the model will have an altered prediction of where the limb will be in space. In effect, the act of adapting alters the model's perception of the limb. Interestingly, there is a growing body of experimental evidence for this same effect. In particular, the act of adapting to a visuomotor disturbance biases the perception of subjects' movement and hand position in a manner consistent with our model [36], [37]. This perceptual bias was found to be nearly half of the adapted rotation, also consistent with our model. Importantly, this bias was found to be associated with the limb alone, and not the result of a global recalibration of visual space [36]. A similar finding demonstrates that adapting to a force field alters the perception of the limb in space as well [38]. On the whole, these results suggest a further link between our model's use of body and world parameters and how the nervous system adapts to new motor behaviors.

Some of our results on interference rely on the relative duration of the counter disturbance, behavior B, in the A-B-A paradigm. Since both disturbances are presented for the same length of time, the counter disturbance almost completely degrades any estimate of the world parameter estimate. This results in motor patterns consistent with interference. If the counter disturbance was presented for approximately twice as long, our model predicts that the typical pattern of interference would not be observed. Rather than producing errors similar to naïve subjects, our model predicts subjects should produce larger errors, consistent with the expectation of the counter disturbance. As far as the authors are aware, this particular experimental result has not yet been performed and would be particularly informative.

In this study we have implicitly assumed that savings is a form recall; previously adapted information is called upon resulting in reduced motor errors relative to naïve conditions. However, other researchers have asserted that savings could be a form of meta-adaptation instead, wherein adaptation rates are facilitated and motor errors decrease faster than during naïve conditions [e.g. 8,39]. By the same token, interference could either be a form of re-adaptation and hijacking of previously adapted behaviors (as we have assumed), or an inability to recall previously adapted information. To the best of the authors' knowledge, both of these options for savings and interference are consistent with the known empirical evidence. However, our model does make some predictions that might speak to these possibilities. For example, our model predicts that on repeated days of training, the estimate of a world-imposed disturbance progressively increases. Assuming cues such as the experimental apparatus are salient for estimating relevance, each day's initial errors should be smaller than the previous. This implies that subjects should eventually display “one-shot” learning of a disturbance. This would be strong evidence that subjects were in fact recalling knowledge, rather than nearly instantaneously adapting. Future studies could examine a similar line of predictions to distinguish between savings as recall, and savings as meta-adaptation.

Relevance as we have defined it here is a relatively simplistic indication of the motor system's current operating condition, or context. Clearly there is more to context than motor errors. For instance, whether or not one is holding the handle of a robot is a clear indicator of the kind of disturbances one might expect [20]. Similarly, while they may not be as salient, cues such as tones and colors may also serve for disambiguating context [40]. Finally, in this study we have completely neglected forces, both the contact forces between the limb and the robot handle, and the forces required to produce movements. This is clearly an oversimplification and it is known that these forces are relevant when adapting [e.g. 41,42,43]. Why some cues are easy to indicate context and others are difficult remains and open question. Which variables the nervous system uses to distinguish context are similarly unknown. We expect that future studies will shed more light on these issues.
